# Validation of the Anticolitis Efficacy of the Jian-Wei-Yu-Yang Formula

**DOI:** 10.1155/2022/9110704

**Published:** 2022-08-31

**Authors:** Jing Yan, Yan Tang, Wei Yu, Lu Jiang, Chen Liu, Qi Li, Zhiqiang Zhang, Changlei Shao, Yang Zheng, Xihao Liu, Xincheng Liu

**Affiliations:** Department of Physiology, Jining Medical University, Jining City, Shandong Province, China

## Abstract

**Background:**

Inflammatory bowel disease (IBD) is a major cause of morbidity and mortality due to its repetitive remission and relapse. The Jian-Wei-Yu-Yang (JW) formula has a historical application in the clinic to combat gastrointestinal disorders. The investigation aimed to explore the molecular and cellular mechanisms of JW.

**Methods:**

2% dextran sodium sulfate (DSS) was diluted in drinking water and given to mice for 5 days to establish murine models of experimental colitis, and different doses of JW solution were administered for 14 days. Network pharmacology analysis and weighted gene co-expression network analysis (WGCNA) were utilized to predict the therapeutic role of JW against experimental colitis and colitis-associated colorectal cancer (CAC). 16S rRNA sequencing and untargeted metabolomics were conducted using murine feces. Western blotting, immunocytochemistry, and wound healing experiments were performed to confirm the molecular mechanisms.

**Results:**

(1) Liquid chromatography with mass spectrometry was utilized to confirm the validity of the JW formula. The high dose of JW treatment markedly attenuated DSS-induced experimental colitis progression, and the targets were enriched in inflammation, infection, and tumorigenesis. (2) The JW targets were related to the survival probability in patients with colorectal cancer, underlying a potential therapeutic value in CRC intervention. (3) Moreover, the JW therapy successfully rescued the decreased richness and diversity of microbiota, suppressed the potentially pathogenic phenotype of the gut microorganisms, and increased cytochrome P450 activity in murine colitis models. (4) Our *in vitro* experiments confirmed that the JW treatment suppressed caspase3-dependent pyroptosis, hypoxia-inducible factor 1*α* (HIF1*α*), and interleukin-1b (IL-1b) in the colon; facilitated the alternative activation of macrophages (M*φ*s); and inhibited tumor necrosis factor-*α* (TNF*α*)-induced reactive oxygen species (ROS) level in intestinal organoids (IOs).

**Conclusion:**

The JW capsule attenuated the progression of murine colitis by a prompt resolution of inflammation and bloody stool and by re-establishing a microbiome profile that favors re-epithelization and prevents carcinogenesis.

## 1. Introduction

Inflammatory bowel disease (IBD) is a complex set of diseases that account for the major cause of morbidity and mortality, with an increasing incidence rate worldwide. IBD features repetitive remission and relapse due to a broad range of causal and synergistic factors such as genetic susceptibility, inappropriate diet, and environment, as well as microbial dysbiosis. The host hallmarks of IBD encompass persistent barrier dysfunction, dysbiosis, and abnormal intestinal epithelial cell death, all of which are associated with overactive immune status, with the consequent complications including colitis-associated carcinogenesis (CAC). The ultimate therapeutic strategy for IBD management is the success in re-epithelialization without unstrained proliferation and cell transformation, which relies on the recovery of gut homeostasis including healthy microbiota communities, maintenance of intestinal stem cell niche, and orchestrated host immune system [1]. Therefore, multitarget drugs exhibit stronger clinical efficacy than exquisitely selective compounds. Derived from the oldest Chinese medical book “Shang Han Lun” and evolving with the modern medical technique, traditional Chinese medicine (TCM) has a long historical application in the clinic and is a golden resource for developing multitarget drugs. However, the pharmacological mechanisms of many efficacious drugs need extensive experimental evidence to corroborate the efficacy and guarantee low toxicity. The emergence of system pharmacology and omics technology helps resolve the difficulty in explaining the synergistic and counteracting effects of multiple herbs included in each TCM formula, providing a chance for the development of TCM.

An efficacious strategy to interfere with the progression of UC is not only limited to the resolution of inflammation and mucosal injury but can also restore the homeostasis of gut functions, namely, a healthy microbiome profile and accommodative immune system, as well as the prevention of complications. To this end, a formula, composed of multiple natural medical herbs functioning synergistically and counteracting thus to reduce toxicity and enhance efficacy, is a promising alternative therapy to combat UC. The Jian-Wei-Yu-Yang (JW) formula, an officially listed formula in the Chinese Pharmacopoeia 2020, is composed of *Bupleurum chinense* DC. (BD), *Paeonia lactiflora* Pall. (PP), *Corydalis yanhusuo* (Y. H. Chou & Chun C. Hsu) W. T. Wang ex Z. Y. Su & C. Y. Wu (CWT), *Codonopsis affinis* Hook. f. & Thomson (CH), *Bletilla chartacea* (King & Pantl.) Tang & F. T. Wang (BT), *Strobilanthes cusia* (Nees) Kuntze (SK), *Glycyrrhiza uralensis* Fisch. (GL), and pearl powder. In this formula, several anti-inflammatory ingredients are included, namely, CH [2, 3], LC [4, 5], SK [6, 7], BT [8–10], BD [11–13], PP [14, 15], and CWT [16–18], suggesting potent therapeutic value when combating colitis.

The present study delineated the relevant pharmacological mechanisms by multiomics integrated with network pharmacology and corroborated the efficacy against colitis through *in vitro* and *in vivo* experiments.

## 2. Materials and Methods

### 2.1. Ethics Statement

Experiments were conducted under the supervision of the guidelines of the Institutional Animal Care and Use Committee of Jining Medical University in China (SYXK-Shandong province-2018-0002).

### 2.2. Network Pharmacology

A JW-component-target network was constructed by active components with a cutoff of drug-likeness (0.18) and oral bioavailability (20%). All the plant names have been checked with https://www.theplantlist.org. The targets were obtained from PubChem, and the genes of ulcerative colitis (UC) were collected from GeneCards [19], Online Mendelian Inheritance in Man (OMIM) [20], DrugBank [21], PharmGKB [22], and Statistics of Therapeutic Target Database (TTD). Based on the commonly shared genes, Gene Ontology (GO) and Kyoto Encyclopedia of Genes and Genomes (KEGG) Pathway Enrichment were calculated, respectively [23]. Herb-Ingredient-Target (HIT) Interaction network and a protein-protein interaction (PPI) network with a confidence score ≥0.7 based on the Search Tool for Retrieval of Interacting Genes/Proteins database were computed by CytoNCA [24], which calculates the median values of betweenness centrality (BC), closeness centrality (CC), degree centrality (DC), local average connectivity (LAC), eigenvector centrality (EC), and network centrality (NC).

### *2.3*. Clinical Patient Data Acquisition and Weighted Gene Co-Expression Network Analysis (WGCNA)

A total of 426 patients with colon adenocarcinoma (COAD) with the clinical information were obtained from The Cancer Genome Atlas (TCGA) database. To identify the role of JW in CAC, the mRNA expression of the targets of JW was calculated utilizing the “limma” package, and “ConsensusClusterPlus” package was applied to divide the tumor samples into 2 clusters. Utilizing *R* “survival” and “survminer” [25] packages, the Kaplan–Meier (KM) plotter showed the overall survival probability [26]. A Cox proportional hazard model was established to indicate JW targets associated with the survival probability in COAD patients. Hazard ratio (HR) with 95% confidence intervals and log-rank *p* value were calculated via univariate survival analysis, and a *p* value of <0.05 was considered statistically significant. By the *R* package “glmnet,” the OS-related JW genes in univariate Cox regression analysis were then included to perform in least absolute shrinkage and selection operator (LASSO) regression analysis. The penalty regularization parameter (*λ*) was calculated to prevent overfitting effects. According to the optimal lambda value and the corresponding coefficients, 15 JW genes were selected to establish the risk signature. Patients were clustered into two groups: low- and high-risk groups by the average risk score, and KM analysis evaluated the survival between high- and low-risk groups. Receiving operating characteristic (ROC) curve with the area under the curve (AUC) was computed by “pROC” and “ggplot2” packages. AUC represents the degree or measure of separability as a value between 0 and 1 and was utilized to evaluate the discrimination ability of the model. The higher is the better. ROC curve was constructed by true positive rate (TPR) and false positive rate (FPR). To determine the associated immune cell effector functions with the JW targets, “GSVA” package was used for single-sample gene set enrichment analysis (ssGSEA).

### 2.4. JW Formula Preparation and Identification

30 g JWYY formula (Table 1) was prepared by mixing the powder of CH (20.85 g), BD (20.85 g), BT(20.85 g), PP(20.85 g), CWT(20.85 g), SK(6.25 g), GL(6.25 g), and pearl powder (6.25 g). Briefly, CH, BD, BT, PP, CWT, and GL were boiled and concentrated, and the SK and pearl powder were added. To validate the components in the formula, a connected system of LC-30 (Shimadzu)-hybrid quadruple time-of-flight mass spectrometer (TOF-MS) with electrospray ionization source (ESI) was used. InertSustain C18 column (Shimadzu, 100 × 2.1 mm, 2 *μ*m) was used to perform chromatographic separation with a flow rate of 0.3 ml/min at 35°C. Mobile phase system was composed of equate A (acetonitrile) and equate B (0.1% HCOOH-H_2_O): 4 min (A: 5% : B: 95%), 8 min (A: 20% : B: 80%), 2 min (A: 15% : B: 75%), 2 min (A: 46% : B: 54%), 3 min (A: 100% : B: 0%), and 1 min (A: 5% : B: 95%). Data were acquired in information-dependent acquisition (IDA) with high sensitivity mode, collision energy was 35 ± 15 eV, and declustering potential was ±60 V (Supplementary Table 1) (Supplementary Figure 1).

### 2.5. Serum Isolation

After the high dose treatment of JW, we collected the serum from colitis mice in two hours for the following experiments. To examine the toxicity, NCM460 cells, an epithelial cell line derived from the normal colon of a 68-year-old Hispanic male, were treated with the serum and subjected to an MTT assay (3-[4, 5-dimethylthiazol-2-yl]-2,5 diphenyl tetrazolium bromide) and cellular immunofluorescence assay with BAX antibody (Abcam, USA).

### 2.6. Experimental Colitis Murine Models with the Administration of JW

Mice (Pengyue animal center of Shandong province, China) (male, about 22 g, 2 months old) were given 2% dextran sodium sulfate (DSS, MP Biomedicals) for 1 week, and then the JW was administered for 2 weeks [27]. The dose of the human being is 120 mg/kg per day based on the Chinese Pharmacopoeia 2020, and correspondingly murine is 1.44 g/kg calculated by the Meeh-Rubner formula. 36 mg (equal to 1.44 g/kg) or 18 mg (equal to 0.7 g/kg) JW diluted in 200 *μ*l water was administered each day. The positive control of the study is mesalazine (MedChemExpress, China) [28]. Every 15 mice were grouped, and five groups were set as follows: 0.9% saline, 2% DSS, low-JW-DSS group (5 g/kg), and high-JW-DSS group (10.5 g/kg), mesalazine group (200 mg/kg). Isoflurane (RWD life science, Shenzhen city, China) was used for gas anesthetization. Histopathological score was determined: weight loss (normal: 0; <5%: 1; 5∼10%: 2; 10∼20%: 3; >20%: 4); feces (normal: 0; soft: 1; very soft: 2; and liquid: 3); blood test (no blood within 2 min: 0; positive in 10 s: 1; light purple within 10 s: 2; and heavy purple within 10 s: 3) (Leagene, China); and histological indices (destruction of the epithelium, immune cells infiltrates, edema, crypt loss, each for 1).

Colon samples were soaked in paraformaldehyde (PFA) for 72 hours, embedded in paraffin and then cut into 3 *μ*m thick slices, and subjected to hematoxylin and eosin (H&E) staining.

### 2.7. Enzyme-Linked Immunosorbent Assay (ELISA)

ELISA kits were bought from Abcam and utilized to examine the cytokine concentrations in the serum: TNF*α* (sensitivity: 0.1 pg/ml, range: 47 pg/ml-3000 pg/ml) and IL-1b ELISA Kits (sensitivity: 15.2 pg/ml, range: 28.1 pg/ml-1800 pg/ml).

### 2.8. Amplicon Sequencing Data Analysis

The feces and mucus within the colon were collected and mixed and processed by 16S rRNA sequencing. Raw data were filtered [29] and produced paired-end reads. Corrected paired-end reads generated circular consensus sequencing (CCS) reads. After removing chimeras, OTU (operational taxonomic unit) analysis was performed with a similarity >97%. Krona, as a powerful metagenomic visualization tool, demonstrates species annotation. Each fan means the corresponding annotation's proportion [30]. PICRUSt (Phylogenetic Investigation of Communities by Reconstruction of Unobserved States) analysis was conducted to predict the alterations in KEGG pathways. The raw data are available with the accession number in SRA (PRJNA827781).

### 2.9. Untargeted Metabolomics

Feces and mucus samples were treated with methanol and standard internal substances. After the ultrasound and frozen treatment, the samples were centrifuged. Supernatant was subjected to ultra-high-performance liquid chromatography (UHPLC) coupled with TOF-MS [31]. Acquisition software (Analyst TF 1.7, AB Sciex) was utilized to assess the full scan survey MS data, which were then converted by ProteoWizard and generated the retention time (RT), mass-to-charge ratio (*m*/*z*) values, and peak intensity. According to the in-house MS2 database, substances were identified and determined by orthogonal projections to latent structures-discriminant analysis (OPLS-DA). The cutoff is *p* value < 0.05, fold change (FC) >1.5, and variable importance in the projection (VIP) >1 [32]. Human Metabolome Database (HMDB) [33] and KEGG database were utilized to annotate metabolites.

### 2.10. Peritoneal Macrophage (M*φ*) Isolation

Mice were anesthetized, and sterilized PBS was injected into the abdominal cavity. After massage, the liquids were isolated and centrifuged. The pellet was diluted in 1640 complete medium.

### 2.11. Wound Healing Assay

A density of 10^5^ NCM460 cells was seeded in the 24-well cell culture plate at 37°C in a 5% CO_2_ incubator. When reaching 100% confluency, a scratch was made, and a transwell insert (Corning, 0.3 *μ*m diameter, USA) carrying 10^4^ peritoneal M*φ*s was placed. After 24 hours, cells in 5 random fields were examined.

### 2.12. Western Blotting

Total protein (40 *μ*g) was subjected to SDS-PAGE (sodium dodecyl sulfate-polyacrylamide gel electrophoresis) and transferred. After blockage, antibodies (CASP3, HIF-1*α*, IL-1b, IL-10, and NOS2) were incubated with the membrane for 24 hours. After washing with PBS, secondary antibodies conjugated with horseradish peroxidase (HRP) were utilized, and an enhanced chemiluminescence (ECL) substrate was added to visualize the protein bands. All chemicals were bought from Invitrogen, USA.

### 2.13. Intestinal Organoid Culture

The small intestine was cut into small pieces and washed with cold PBS until the solution became clear. After 35 minutes of digestion by digestive solution [34], the filtered medium was centrifuged and cultured in growth medium. After 9 days, tumor necrosis factor-*α* (TNF*α*) was added with or without JW serum. After 24 hours, IOs were incubated at 37°C with propidium iodide (PI)/Hoechst 33342 (480 nm/630 nm) for 20 minutes. To measure mitochondrial stress, MitoSOX™ Red Mitochondrial Superoxide Indicator (590 nm) was added. After 10 minutes at 37°C, cells were examined using a fluorescent microscope. Two dyes and culturing reagents were bought from Thermo Fisher and STEMCELL Technologies, respectively.

### 2.14. Statistical Analysis

Data are means ± SEM. An unpaired Student's *t*-test was used to compare the differences between the two groups.

## 3. Results

### 3.1. Therapeutic Role of the JW Formula against Experimental Colitis

**Figure 1:**
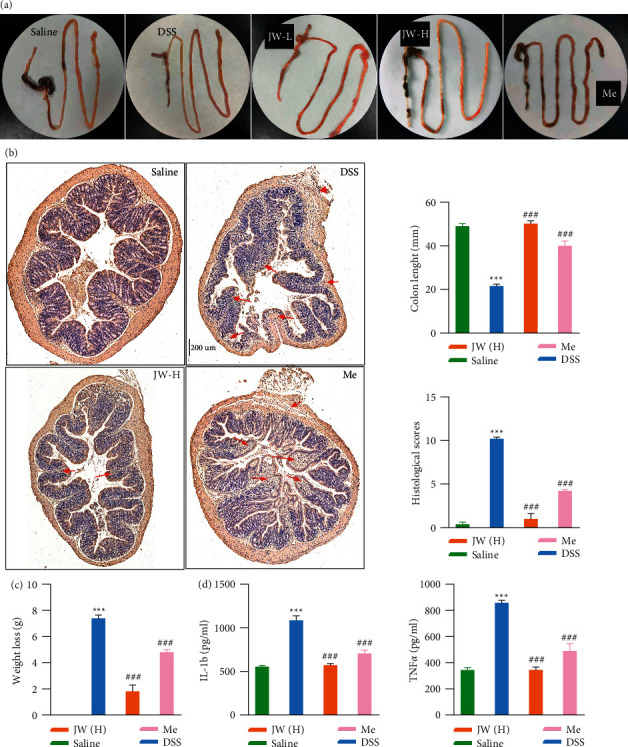
JW formula alleviates the progression of experimental colitis. Colon tissue (a), HE staining of the colon (b), and (c) colon length, weight loss, histological scores, serum TNF*α*, IL-17, and IL-1b levels in ulcerative colitis (UC) murine models after the JW treatment. ^*∗∗∗*^*p* < 0.001 indicates a statistically significant difference from the saline group; ^##^*p* < 0.01 and ^###^*p* < 0.001 indicate a statistically significant difference from the UC group.

We determined the chemical profile of the JW formula by UPLC-MS/MS and identified the presence of the ingredients (Supplementary Figure 1) (Supplementary Table 1). JW was administered to mice subjected to experimental colitis for 14 days. DSS treatment caused weight loss, bloody stool, and hyperemia of the appendix in the murine models of experimental colitis, concomitant with a shortened colon length. These symptoms were markedly alleviated by the JW therapy at a low as well as high dose. As compared with the mesalazine treatment, the high dose of JW produced a longer colon (Figure 1(a)). H&E staining showed that JW successfully rescued colitis-caused crypt loss, alleviated epithelial breach, as well as immune cell infiltrates, showing its superiority in mucosal healing and resolution of inflammation compared with mesalazine (Figure 1(b)). Of note, both doses of JW suppressed the DSS-evoked IL-1b and TNF*α* levels (Figure 1(c)).

### 3.2. Network Pharmacology and WGCNA

**Figure 2:**
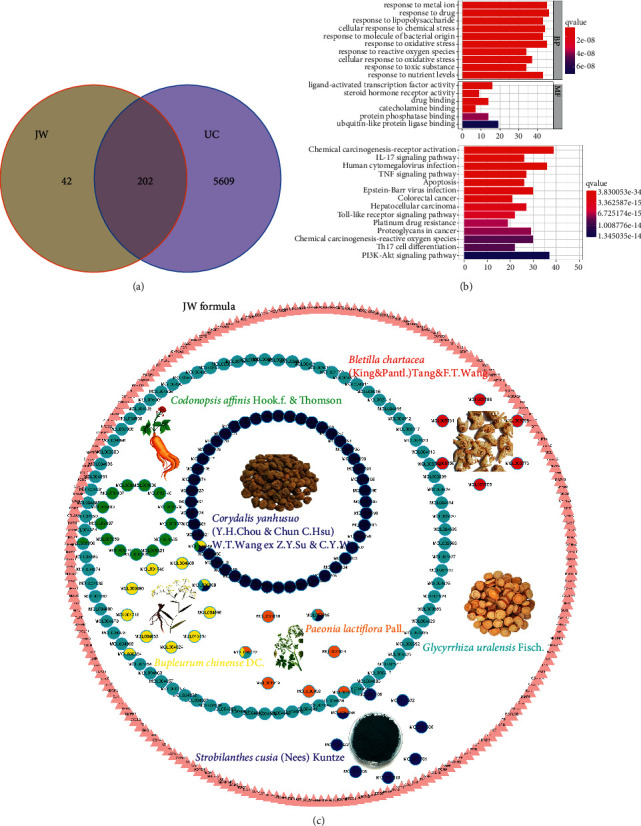
Component-target network of JW. Venn plot illustrates the shared targets between JW and UC (a); GO and KEGG analyses of the shared genes (b); the network of compounds and the targets in UC (c).

**Figure 3:**
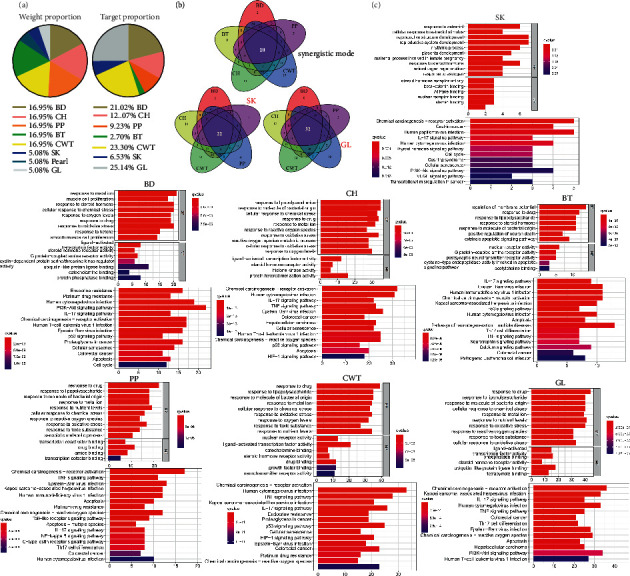
Pharmacological mechanisms of JW. Pie chart demonstrates the percentage of the target genes and weight percentage of JW ingredients (a); GO (upper) and KEGG (lower) analyses show the predicted function of JW components (b).

To predict the pharmacological mechanism of the JW formula, network pharmacology was performed. The JW formula encompassed 8 herbs and shared 202 putative targets with the acquired 5811 colitis-relevant genes (Figure 2(a)) (Supplementary Table 2). An HIT network representing the interactions between active components and targeted genes was established: purple rectangle nodes represented colitis-associated genes, and nodes with different colors inside were active components (Figure 2(c)). GO and KEGG analyses demonstrated that these shared genes were enriched in inflammatory responses, carcinogenesis, infections, and pathogen-recognition pathways including IL-17, TNF signaling pathways, CRC, and response to the bacterial origin, reactive oxygen species (ROS), and lipopolysaccharide and drug (Figure 2(b)). As illustrated in the pie charts, weight or target proportion, BD, CH, PP, and CWT occupied much of the formula (Figure 3(a)). Venn charts demonstrated the shared targets among the ingredients (Figure 3(b)). All the ingredients are associated with infection and CRC, as well as inflammatory responses (Figure 3(c)).

**Figure 4:**
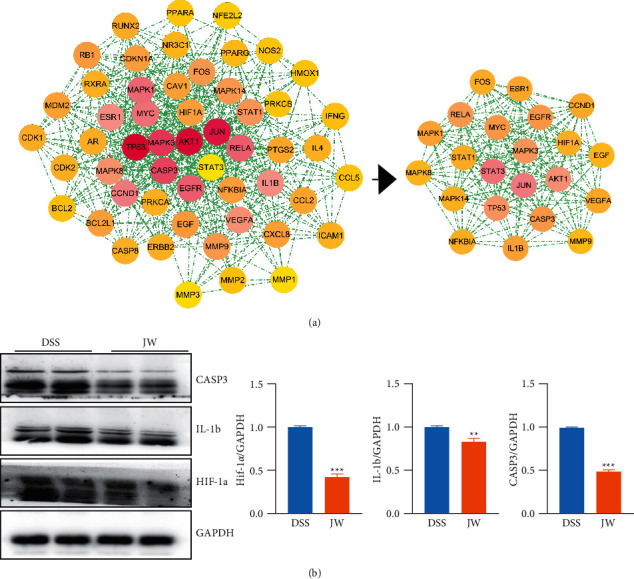
Protein-protein interaction network of JW formula. Interaction network (a); the expression of CASP3, PPAR-*α*, and IL-1b in colon samples from colitis mice with or without JW treatment (b). ^*∗∗∗*^*p* < 0.001 indicates a statistically significant difference from the saline group.

Based on the shared genes between colitis and the JW formula, a PPI network with a PPI enrichment *p* value (<1.0*e*–16) was established utilizing the STRING database, and a subnetwork composed of 59 genes is shown in Figure 4(a). A final network with 20 hub genes was established with the median values (BC: 18.3018139, CC: 0.604651163, DC: 18, EC: 0.119857475, LAC: 11.3, and NC: 12.82258772). Western blotting confirmed that the JW treatment significantly suppressed the protein abundance of CASP3, IL-1b, and HIF-1*α* expression (Figure 4(b)), indicating an alleviated inflammation by the JW formula. Moreover, a hub gene-component network was constructed to predict the major active components of the JW formula (Supplementary Figure 2).

**Figure 5:**
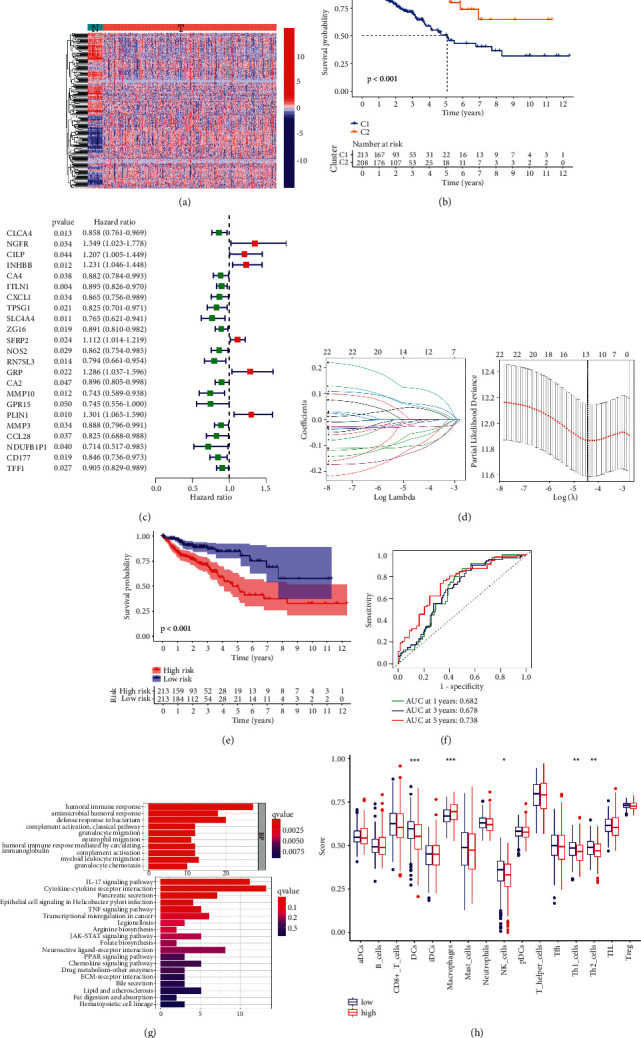
Therapeutic value of JW capsule in CRC. Kaplan–Meier curves demonstrating the prognostic value of clusters in patients with COAD (a); uni-Cox analysis showing the hazard ratio of JW targets (b); Lasso model establishment (c, d); Kaplan–Meier curves indicating the correlation of survival with high-risk, and low-risk groups (e); ROC curve demonstrating the sensitivity and specificity of Lasso model (f); PCoA plot showing the clusters of groups (g); KEGG analysis of the genes in the model (h).

Given the risk of developing cancer due to a suppressed immunity, WGCNA was performed based on TCGA database. Among the targets of all the ingredients, there were 210 differentially expressed genes between patients with COAD and healthy cohorts (Figure 5(a)) (Supplementary Table 3). A total of 426 patients with COAD were divided into two clusters utilizing consensus clustering, which correlated with the survival probability (Figure 5(b)). Univariate Cox regression analysis yielded 23 JW-targeted genes associated with the survival in COAD patients (Figure 5(c)), and the least absolute shrinkage and selection operator (Lasso) regression analysis selected 13 genes as prognostic signatures (*TPSG1*, *ITLN1*, *INHBB*, *SLC4A4*, *SFRP2*, *RN7SL3*, *GRP*, *MMP10*, *PLIN1*, *MMP3*, *NDUFB1P1*, *TFF1*, and *CD177*) (Figure 5(d)) (Supplementary Table 4). KM plot showed that the high-risk group had a lower survival rate than the low-risk group (Figure 5(e)). The prognostic value was corroborated by 1-, 3-, and 5-year survival rates. The AUC values were 0.682, 0.678, and 0.738, respectively (Figure 5(f)), indicating a favorable discrimination performance for the outcome prediction. The KEGG analysis confirmed its antineoplastic effect involving arginine biosynthesis, TNF, and PPAR signaling pathways (Figure 5(g)), and ssGESA demonstrated that the JW targets were associated with antigen-presenting cell functions including dendritic cells and macrophages (Figure 5(h)). Therefore, macrophages were chosen for further *in vitro* experiments.

### 3.3. Fecal Microbiota and the Metabolites

**Figure 6:**
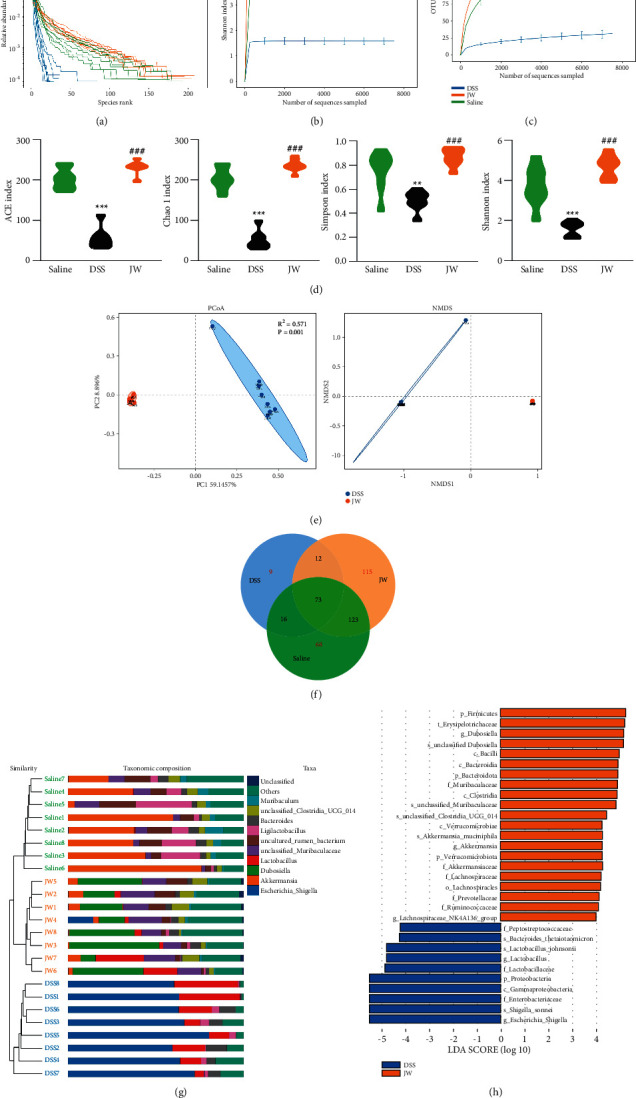
Alteration in microbiota profile in UC murine models after JW treatment. Alpha diversity of microbial communities in mice undergoing UC and treated with JW (a). PCoA (b) and NMDS (c) show the clustering of gut flora; weighted uni-Frac distance with PERMANOVA (d) and a heat map (e) indicating the clusters of saline, DSS, and JW-treated groups at the genus and phylum levels, respectively; LEFSe (f) illustrates the abundance of bacterial species in UC murine models after JW administration. ^*∗∗*^*p* < 0.01 and ^*∗∗∗*^*p* < 0.001 indicate a statistical difference from the saline group; ^###^*p* < 0.001 indicates a difference from the UC group.

Given that dysbiosis is closely associated with the initiation and progression of colitis, 16S rRNA sequencing was conducted utilizing the feces and mucus from mice subjected to experimental colitis with or without the JW therapy. All sample libraries covered >99%, suggesting a sufficient library size to represent most microbes (Supplementary Table 5). Shannon curves with flat ends and rarefaction analysis demonstrated the sufficient OTU numbers for covering the majority of species and saturation, and the rank abundance curve ranking the OTU abundance of each sample by size indicates the richness and evenness of the species. The wider curve means a more abundant species composition, and the flatter shape represents a more even species (Figures 6(a)-6(c)). DSS treatment led to a marked decrease in the alpha diversity of microbiota including Chao and Ace, and Shannon as well as Simpson, all of which were reversed by the JW therapy (Figure 6(d)). In addition to the shared 73 OTUs among the saline, DSS, and JW groups, there were 115 OTUs assigned to the JW group (Figure 6(f)). *β*-Diversity was calculated by binary Jaccard method. Principal Co-ordinates Analysis (PCoA) combined with PERMANOVA showed that JW treatment significantly altered the *β*-diversity of commensal microorganisms (Figure 6(e)). Nonmetric multidimensional scaling analysis (NMDS) indicated the JW-DSS group clustered distinctly from the DSS group with a stress value of 0.02 (Figure 6(e)). Weighted uni-Frac distance with PERMANOVA and a heat map indicated a clear distinction among the three groups (Figure 6(g)). Linear discriminant analysis coupled with effect size measures (LEFSe) showed that the JW treatment increased the relative abundance of *Bacteroidota*, *Verrucomicrobiota,* and *Firmicutes*, whereas it decreased the prevalence of *Proteobacteria* at the phylum level. The administration of JW suppressed the abundance of *Lactobacillus*, *Escherichia, and Shigella*, while increased *Lachnospiraceae NK4A136* and *Akkermansia muciniphila* (Figure 6(h)). BugBase feature prediction calculated by Mann–Whitney–Wilcoxon analysis showed that JW treatment significantly reversed DSS-induced abundance of potentially pathogenic microorganisms and formed biofilm phenotype microorganisms (Supplementary Figure 3A). PICRUSt analysis demonstrated that DSS enhanced carbohydrate, amino acids, lipid, and energy metabolism; increased the risk of developing immune diseases, infections, and cancers, as well as xenobiotics metabolism; and exacerbated drug resistance, while the administration of JW abolished these pathways, as evidenced by the inhibited infections and cancers (Supplementary Figure 3B).

**Figure 7:**
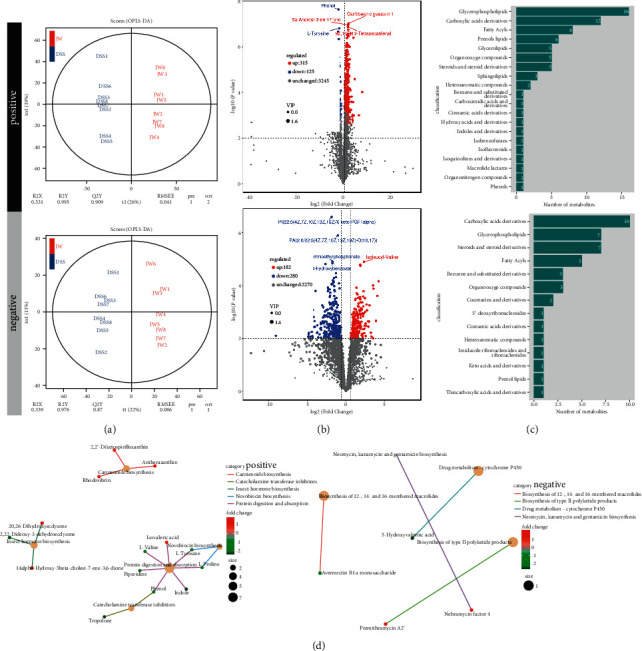
Untargeted metabolomics of UC murine models after JW treatment. OPLS-DAOPLA-DA (a) and volcano chart (b), HMDB enrichment (c), and KEGG pathways (d) present the clusters of gut microorganisms between UC- and JW-treated UC mice under positive and negative modes.

OPLS-DAOPLA-DA combined with a permutation test also validated the differential metabolites between the DSS and DSS-JW groups with Q2Y (0.909, 0.976) and R2Y (0.995, 0.976) (Figure 7(a)). The top 5 regulated metabolites are listed in Figure 7(b) (Supplementary Table 7). The differential metabolites between JW and DSS groups were associated with carboxylic acids and derivatives, glycerophospholipids, fatty acyls, and steroids and derivatives (Figure 7(c)). As compared with the DSS group, the administration of JW inhibited drug metabolism and enhanced the biosynthesis of neomycin, kanamycin, and gentamicin (Figure 7(d)), suggesting that the JW formula could suppress the metabolism of the drugs, and hence increase the drug availability, and inhibit infection.

### 3.4. JW Formula Improves the Survival of Intestinal Stem Cell

**Figure 8:**
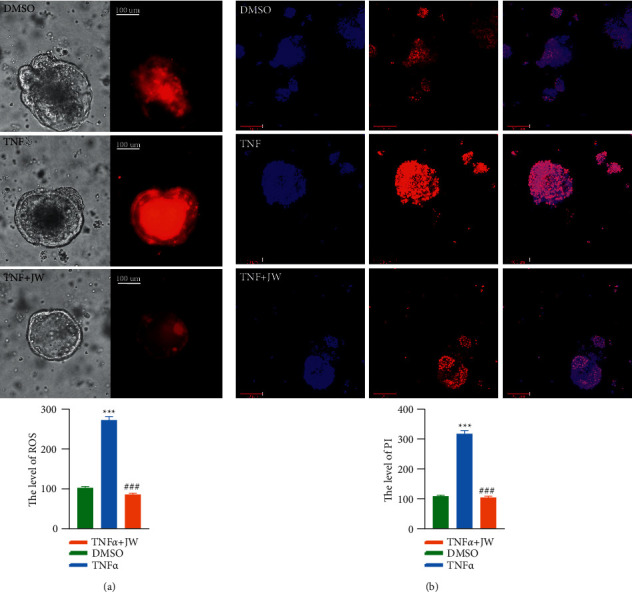
JW serum rescues inflammation-induced cell death of intestinal organoids. The mitochondrial stress (a) in TNF*α*-treated intestinal organoids with JW serum for 24 hours; Hoechst 33342/PI staining (b). ^*∗∗∗*^*p* < 0.001 indicates a statistical difference from the DMSO group; ^#^ indicates a difference from the TNF*α* group.

During colitis, excessive ROS in mitochondria leads to cell death, such as apoptosis or pyroptosis. Dysregulation of cell death, especially the instigated pyroptosis of intestinal stem cells (ISCs), exacerbates inflammation and hampers mucosal healing. IOs were cultured as a model to evaluate the proliferation of ISCs. We treated IOs with TNF*α* (20 ng/ml, 24 h) to construct an *ex vivo* UC cellular model. Serum was isolated from murine colitis models subjected to the JW treatment as well as healthy blank controls, and the JW serum did not influence BAX expression and the viability of NCM460 cells (Supplementary Figure 4). TNF*α* treatment swelled IOs and increased the ROS level, and this elevation was diminished by JW serum (Figure 8(a)). Moreover, TNF*α*-induced IO death was rescued after the administration of JW serum (Figure 8(b)).

### 3.5. JW Contributes to the Alternative Activation of M*φ*s

**Figure 9:**
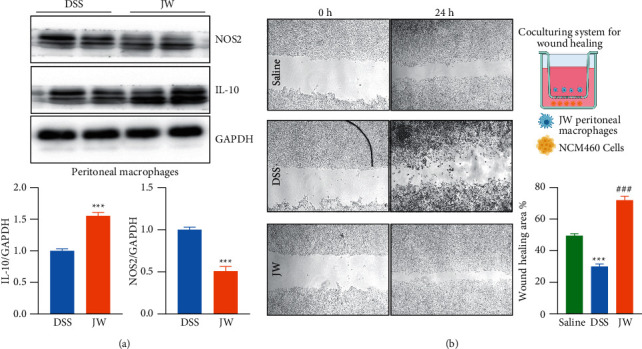
JW facilitates the alternative activation of macrophages. The protein abundance of NOS2 in peritoneal macrophages from UC murine models after JW treatment (a); wound healing assay (b) showing the migration of NCM460 cells in the presence of peritoneal macrophages from the JW group. ^*∗∗∗*^*p* < 0.001 indicates a statistical difference from the saline group, and ^#^ indicates a difference from the DSS group.

Peritoneal M*φ*s were collected from UC murine models. The JW formula suppressed the protein abundance of NOS2 and increased IL-10 expression, suggesting an enhanced M2 transition (Figure 9(a)). Moreover, NCM460 cells were co-cultured with the JW peritoneal M*φ*s for 24 hours. The wound healing experiment showed an increased migration in the JW group, confirming the favored wound healing capacity of M2 M*φ*s (Figure 9(b)).

## 4. Discussion

In the present study, we utilized systematic pharmacology, 16S rRNA sequencing integrated with untargeted metabolomics, and biochemical experiments to discuss the molecular and cellular mechanisms of the JW formula and evaluate its efficacy against experimental colitis.

Ulcerative colitis is a subtype of IBD and occurs mainly in the colon due to a broad spectrum of factors including the microbiome, genetic susceptibility, diet, host immune status, and environment. Patients with UC suffer from mental disorders and physical disadvantages, for example, rectal bleeding, abdominal pain, malnutrition, and diarrhea, with a higher risk of developing CAC than non-UC cohorts [35].

In the JW formula, BD, CH, PP, BT, and CWT were equally responsible for 84.75% of the weight, while BD, CH, and CWT shared most targets. We calculated the common targets among these ingredients and observed the least shared genes when combined with BT, indicating that BT functions independently to a certain extent. All 7 ingredients were involved in infections and cancers and inflammatory responses such as IL-17, TNF, and HIF-1 signaling pathways, which was corroborated by our *in vivo* experiments. We also observed a more prominent anticolitis effect of the JW formula on the colitis progression than that of mesalazine, which is a common drug to treat colitis.

During the progression of UC, the microbiota profile and host metabolic pathways change in response to the stimulation of proinflammatory cytokines and pathogens as well as their metabolites, and these alterations further exacerbate the compromised gut functions leading to severe symptoms. Successful therapy should not only be able to timely stop hemostasis and pain and rescue the impaired gut barrier integrity but also need to re-establish the homeostasis of the microbiome and promote the resolution of inflammation in a short span of time. Here, we delineated the pharmacological mechanism of the JW therapy from multiple angles, namely, prompt resolution of physical and mental suffering, microbiome, drug metabolism, inflammation, and complications:

- Bloody stool and pain: low gastrointestinal bleeding frequently occurs in patients with UC and exacerbates the pain and mental suffering of patients [36], which is a major cause of morbidity and mortality of the disease. BT not only facilitates blood aggregation and is considered a potent hemostatic agent [37, 38] but can also promote wound healing [10, 39], highlighting the resolution of blood stool and subsequent mucosal healing. Concurrently, the antithrombotic property of CWT [40] resolves the concern regarding blood stasis due to the utilization of antihemorrhagic ingredients. Of note, both of these herbs [41, 42] are also the most widely consumed herbal products for the treatment of pain, which is a challenging problem in IBD treatment due to the concerns regarding the risk of relapse of IBD and injury to the gut mucosa induced by long periods of anodyne administration. Our *in vivo* experiment corroborated that the JW therapy timely stopped the bloody stool and alleviated the weight loss. Collectively, the JW formula could effectively relieve both urgent symptoms, and no extra analgesia is needed.
- Maintaining a healthy microbiome profile: the gut microbiota resides predominantly in the mucus layer of the colon, and its colonization provides substantial protection against exogenous challenges including the invasion of pathogenic bacteria. The composition and diversity of the gut commensal flora exert pivotal roles in orchestrating gastrointestinal functions and gut immunity, and dysbiosis is considered a detrimental factor of UC. In the JW formula, GL [4, 43], CWT [44, 45], BD [46], CH [47, 48], SK [49, 50], and BT [51, 52] could reshape the colonization of the gut microorganisms by their prebiotic-like activities.
- In full accordance with our results, DSS-induced colitis decreases the richness and diversity of the gut microbiota [53], both of which were reversed by the JW formula, highlighting its contribution to the remodeling of gut microbiota. An imbalanced gut commensal flora often begins with an increased prevalence of the *Proteobacteria* and *Bacteroides thetaiotaomicron,* which have been considered potential diagnostic signatures of dysbiosis, depression, and colitis [54, 55], and the JW therapy considerably suppressed their colonization. Consistently, our untargeted metabolomics results showed that the JW therapy enhanced neomycin, kanamycin, and gentamicin biosyntheses, which might be attributable to the suppression of pathogen colonization. Moreover, DSS treatment induced the colonization of the genera *Escherichia and Shigella* which are involved in the development of UC into CAC [56], which were also inhibited by JW treatment. Additionally, the increased relative abundance of *Dubosiella* and *Lachnospiraceae NK4A13* by JW is negatively correlated with the levels of inflammation-promoting cytokines [57] and favors gut barrier integrity [58], respectively. Noteworthy is the facilitated colonization of *Akkermansia muciniphila*, which improves intestinal homeostasis [59, 60], contributes to immune tolerance in response to gut commensal flora [61], and attenuates the symptoms of colitis [62] and metabolic disorders [63]. PICRUSt analysis predicted that the increased risks of developing infections, cancers, and carbohydrate metabolism were suppressed by the JW treatment. Altogether, JW treatment restores the colonization of gut commensal flora and plays a protective role in the gut.
- Restoring the homeostasis of gut immunity and mucosal healing: during colitis, neutrophil infiltrates cause the excessive formation of highly reactive species inducing oxidative stress [64]. Meanwhile, the infiltration of immune cells induces persistent inflammatory reactions and destroys epithelial integrity and the barrier function, which allow for the pathogen invasion and subsequent antibacterial or antivirus immunity, if still exists. Therefore, resolution of inflammation and alleviation of peroxidation are fundamental to treating colitis, which are effectively accomplished by all of the components: CH [2, 3], LC [4, 5], SK [6, 7], BT [8–10], BD [11–13], PP [14, 15], CWT [16–18], and pearl powder [65]. First of all, the JW formula efficaciously alleviated the inflammatory reactions during the progression of colitis, as evidenced by the decreased IL-1b and TNF*α* levels in the serum of colitis mice after the JW therapy. Given the correlation of M*φ* functions and the JW-targets predicted by WGCNA, the phenotypes of peritoneal M*φ*s were examined after the JW treatment. M1 M*φ*s are responsible for initiating innate immunity, while M2 M*φ*s favor the resolution of inflammation and wound healing [66, 67]. We confirmed that the JW treatment enhanced the transition of M2 M*φ*s and hence increased the migration of colorectal epithelial cells. Secondly, the administration of JW markedly inhibited the neutrophil infiltration and CASP3-dependent inflammation-promoting pyroptosis [68, 69] in murine colitis models and suppressed TNF*α*-induced ROS production in IOs. Collectively, the JW formula rescues inflammation-induced pyroptosis and facilitates the resolution of inflammation in the crypt niche where intestinal stem cells predominantly reside. We posit that the anti-inflammation and antioxidation effects of JW formula are highly efficacious. Noteworthy is the immune-enhancement effect of CH [70, 71], which maintains the functional gut immunity for necessary pathogen recognition and elimination.
- Complications: ① a broad array of investigations have reported the antitumor effect of BT [72], PP [73], BD [74–76], CH [77, 78], LC [79, 80], SK [81–83], and CWT [84, 85], which is considered a major complication of UC. Based on TCGA database, the targets of JW clustered COAD patients into two groups and correlated with the survival probability. Moreover, the lasso model constructed by JW targets showed higher specificity and sensitivity in predicting the outcome of COAD patients, which highlights the antineoplastic role of the JW formula. ② Although colitis does not impair liver function, its severity influences the hepatic metabolism [86] by the microbiota-engaged gut-liver axis and impairs nutrient processing and subsequent lipid accumulation and fibrogenesis. Moreover, oral drug administration exacerbates the metabolic burden in the liver, which might result in metabolic disturbances and thereby increasing the susceptibility to carcinogenesis [86]. Issues regarding hepatotoxicity have been resolved by the promoted liver regeneration by BT [87], PP [88], BD [89], CH [90, 91], LC [92, 93], SK [94], and CWT [95]. ③ IBD patients are susceptible to malnutrition including anemia, amino acid, and vitamin deficiency [96], which could be rescued by PP [97] due to its hematopoiesis-promoting effect [98–100] and by the addition of pearl that is produced from shellfish mollusks which contain 20 amino acids and Fe and Mg elements [101]. ④ Additionally, pearl powder [84, 85, 102] and BT [103] are conductive to re-epithelization by activating the transforming growth factor (TGF) signaling pathway, and CH alleviates mucosal injury by increasing mucus production and suppressing gastric acid secretion [104, 105].
- Orchestrating drug metabolism: since most TCM formulas have multiple herbs functioning in a synergistic or counteracting mode to achieve homeostasis, drug interaction is a major challenge for drug efficacy and safety. As seen in our metabolomics analysis, cytochrome P450 (CYPs), enzymes with catalytic activities regulating drug metabolism and drug-drug interactions [106], was inhibited by the JW treatment, suggesting that the JW treatment facilitates the bioavailability of active components, which might be attributable to PP [107], BD [89, 108], and LC [109, 110]. Altogether, maintaining the balance between drug metabolism and bioavailability is the key of this formula to enhance efficacy and reduce toxicity. As seen in the network constructed with the hub genes and their correlated active components, 107 active components were involved and might be the major bioactive components in the formula. Nevertheless, the limitation of the study is the lack of pharmacokinetic study, and further investigations about the key active components are needed.

## 5. Conclusion

We propose that JW is an efficient formula to treat colitis with the virtue of the prompt resolution of bloody stool and pain. The salutary properties of JW in combating UC are not only limited to the potent antioxidant and anti-inflammatory effects but also considerations about the improvement of drug metabolism, recovery of microbiome profile, and protection against complications.

## Figures and Tables

**Table 1:** Jian-Wei-Yu-Yang formula.

Scientific name of the herb	Chinese name	Material	Abbreviation
*Paeonia lactiflora* pall.	Bai Shao	Rootstalk	PP
*Bupleurum chinense* DC.	Chai Hu	Rootstalk	BD
*Corydalis yanhusuo (Y. H. Chou & Chun C. Hsu) W. T. Wang ex Z. Y. Su & C. Y. Wu*	Yan Hu Suo	Rootstalk	CWT
*Codonopsis affinis* Hook. f. & Thomson	Dang Shen	Rootstalk	CH
*Glycyrrhiza* glabra L.	Gan Cao	Rootstalk	GL
Strobilanthes cusia (Nees) Kuntze	Qing Dai		SK
Bletilla chartacea (King & Pantl.) Tang & F. T. Wang	Bai Ji	Rootstalk	BT
Pearl powder	Zhen Zhu Fen		Pearl

## Data Availability

The data used to support the findings of this study are available from the corresponding author upon request.

## Abbreviations

IBD: Inflammatory bowel disease
TCGA: The Cancer Genome Atlas
COAD: Colon adenocarcinoma
CAC: Colitis-associated carcinogenesis
JW: Jian-Wei-Yu-Yang
TCM: Traditional Chinese medicine
PPIs: Protein-protein interactions
UC: Ulcerative colitis
M*φ*s: Macrophages
ISCs: Intestinal stem cells
IOs: Intestinal organoids
KM: Kaplan–Meier
CC: Closeness centrality
BC: Betweenness centrality
DC: Degree centrality
EC: Eigenvector centrality
LAC: Local average connectivity
NC: Network centrality
GO: Gene Ontology
KEGG: Kyoto Encyclopedia of Genes and Genomes
AUC: Area under the curve
PPAR-*γ*: Peroxisome proliferators-activated receptor-*γ*
TNF*α*: Tumor necrosis factor-*α*
ROS: Reactive oxygen species
CASP3: Caspase 3
IL-17: Interleukin-17
IL-1b: Interleukin-1b
HIF-1*α*: Hypoxia-inducible factor 1*α*.

## Supplementary Materials

Supplementary Materials Supplementary Table 1: LC-MS/MS identified active components of JW. Supplementary Table 2: active components and targets of JW. Supplementary Table 3: differentially expressed genes between colorectal cancer patients and healthy cohorts. Supplementary Table 4: Lasso model of the JW target. Supplementary Table 5: alpha diversity indices of the gut microbiota after JW treatment. Supplementary Table 6: the altered metabolites in murine colitis models after JW treatment. Supplementary Figure 1: LC-MS/MS identified JW components. Supplementary Figure 2: the major active components of the JW formula. Supplementary Figure 3: microbial communities in colitis mice after JW treatment. Rank abundance curve (A), Shannon curves (B), rarefaction analysis (C), and the shared OTUs among three groups (D); BugBase predicts the phenotype of microbiota (E), and PICRUSt predicts the altered pathways after JW treatment (F). ^*∗∗∗*^*p*  <  0.001 indicates a statistical difference from the DSS group. Supplementary Figure 4: MTT assay evaluating the toxicity of the JW serum.
