# Supplementary material for: Validation of the Anticolitis Efficacy of the Jian-Wei-Yu-Yang Formula
Source: Evid Based Complement Alternat Med. 2022 Aug 31;2022:9110704. doi: 10.1155/2022/9110704 (PMC9451982; doi:10.1155/2022/9110704)
Supplement: Supplementary Materials — Supplementary Table 1: LC-MS/MS identified active components of JW. Supplementary Table 2: active components and targets of JW. Supplementary Table 3: differentially expressed genes between colorectal cancer patients and healthy cohorts. Supplementary Table 4: Lasso model of the JW target. Supplementary Table 5: alpha diversity indices of the gut microbiota after JW treatment. Supplementary Table 6: the altered metabolites in murine colitis models after JW treatment. Supplementary Figure 1: LC-MS/MS identified JW components. Supplementary Figure 2: the major active components of the JW formula. Supplementary Figure 3: microbial communities in colitis mice after JW treatment. Rank abundance curve (A), Shannon curves (B), rarefaction analysis (C), and the shared OTUs among three groups (D); BugBase predicts the phenotype of microbiota (E), and PICRUSt predicts the altered pathways after JW treatment (F). ∗∗∗p < 0.001 indicates a statistical difference from the DSS group. Supplementary Figure 4: MTT assay evaluating the toxicity of the JW serum. [file 9110704.f1.zip › sTAB2.docx]

|  | Targets of each ingredient in ulcerative colitis | | | | |  |
| --- | --- | --- | --- | --- | --- | --- |
| CH | CWT | SK | BD | BT | PP | GL |
| TYR | E2F1 | CCL5 | HSPB1 | PPARD | SLPI | E2F1 |
| CDK4 | COL3A1 | GABRA1 | E2F1 | PRSS1 | GABRA1 | COL3A1 |
| RXRA | RXRA | IKBKB | IGFBP3 | RXRA | IKBKB | CDK4 |
| IL4 | HIF1A | SLC6A4 | HIF1A | MAPK14 | RXRA | PLB1 |
| ICAM1 | ICAM1 | RXRA | CHUK | ESR2 | SLC6A4 | HIF1A |
| ESR2 | GJB1 | ADRA1A | ICAM1 | DPP4 | ICAM1 | RXRA |
| MMP1 | ESR2 | MAPK14 | ABCG2 | SCN5A | ADRA1A | ICAM1 |
| PGR | DRD2 | PGR | RXRA | ESR1 | CYP3A4 | ESR2 |
| EGFR | KDR | CASP9 | ESR2 | EGFR | MMP1 | KDR |
| MAOA | EGFR | CHRM1 | MMP1 | F7 | STAT1 | EGFR |
| CCNA2 | F7 | SCN5A | STAT1 | KCNH2 | PGR | GSR |
| CA2 | MAOA | KDR | PGR | CCNA2 | CASP9 | F7 |
| XIAP | CCNA2 | CHRNA2 | PSMD3 | PTGS1 | CHRM1 | CCNA2 |
| NOS2 | GNRH1 | KCNH2 | EGFR | AR | PSMD3 | ADIPOR2 |
| MAOB | RUNX1T1 | CCNA2 | RASSF1 | PPARG | SCN5A | RUNX1T1 |
| PRSS1 | CA2 | CDK2 | F7 | CDK2 | CHRNA2 | CA2 |
| TP53 | FOS | CA2 | MMP3 | GSK3B | IL6R | FOS |
| DPP4 | ADRA2C | PTGS2 | KCNH2 | PTGS2 | AKT1 | IRF1 |
| MMP9 | IRF1 | OPRM1 | MAOA | NOS2 | HMOX1 | NOS2 |
| CASP3 | NOS2 | PRKCA | CCNA2 |  | F7 | SELE |
| TOP2A | SELE | NOS2 | RUNX1T1 |  | KCNH2 | CYP19A1 |
| BCL2L1 | VCP | CDKN3 | IL1B |  | INSRR | RUNX2 |
| CASP7 | RUNX2 | MAOB | F3 |  | CYP1B1 | ODC1 |
| AR | ODC1 | CYP1A1 | FOS |  | HAS2 | DPP4 |
| PTGS1 | CASP3 | TNFAIP6 | IRF1 |  | CD14 | CASP3 |
| CDKN1A | DPP4 | PRSS1 | CHEK2 |  | PTGS2 | TOP2A |
| IL2RA | TOP2A | BCL2 | SELE |  | NOS2 | NR1I2 |
| PLAU | NR1I2 | CASP8 | NOS2 |  | DPEP1 | BCL2L1 |
| ACHE | PDE10A | CHRM3 | CXCL8 |  | OPRM1 | CAT |
| NR3C2 | BCL2L1 | ADRB2 | MAOB |  | PRKCA | CES1 |
| GSTP1 | NOS3 | CASP3 | PRSS1 |  | SELE | NOS3 |
| DRD1 | CDKN1A | BAX | BCL2 |  | CYP1A2 | CDKN1A |
| ADRA1B | IL2RA | ESR1 | ODC1 |  | CYP1A1 | IL2RA |
| OPRD1 | CRH | PPARG | RUNX2 |  | MAPK8 | PON1 |
| GABRA1 | PON1 | AR | TP53 |  | VCAM1 | ACHE |
| MMP13 | ACHE | RELA | EGF |  | TNFAIP6 | CXCL2 |
| SLC6A4 | NR3C2 | PTGS1 | NR1I3 |  | PRSS1 | NR3C2 |
| ADRA1A | CXCL2 | AHR | MMP9 |  | BCL2 | CTSD |
| MAPK14 | ADRA1B | PON1 | DPP4 |  | GSTM1 | ADRA1B |
| PCNA | CTSD | ACHE | CASP3 |  | NR1I3 | OPRD1 |
| LTA4H | OPRD1 | CHEK1 | PPARA |  | CASP8 | SERPINE1 |
| MET | SERPINE1 | JUN | OLR1 |  | CHRM3 | SLPI |
| CASP9 | GABRA1 | GSK3B | TOP2A |  | ADRB2 | GABRA1 |
| CHRM1 | CYP3A4 | DRD1 | NR1I2 |  | CDK1 | ADRA1A |
| SCN5A | ADRA1A | ADRA1B | BCL2L1 |  | CASP3 | MAPK14 |
| HMOX1 | MAPK14 | IFNG | ELK1 |  | DPP4 | CYP3A4 |
| MMP2 | LTA4H |  | NOS3 |  | SLC6A2 | LTA4H |
| IL6R | PRKCB |  | AR |  | NR1I2 | LDLR |
| AKT1 | IGF2 |  | PTGS1 |  | ESR1 | STAT3 |
| IL10RA | SCN5A |  | CDKN1A |  | BAX | IGF2 |
| CCNB1 | AKT1 |  | IL2RA |  | CAT | PRKCB |
| ERBB2 | MMP2 |  | E2F2 |  | PPARG | SCN5A |
| INSRR | IL6R |  | COL1A1 |  | AR | MMP2 |
| MDM2 | IL10RA |  | PLAU |  | RELA | IL6R |
| ADRA2A | CCNB1 |  | PON1 |  | PTGS1 | AKT1 |
| NR3C1 | BMPR2 |  | GJA1 |  | AHR | IL10RA |
| CDK2 | INSRR |  | ACHE |  | PON1 | CCNB1 |
| CCND1 | CAV1 |  | CXCL2 |  | ALOX5 | INSRR |
| SLC6A3 | CYP1B1 |  | NR3C2 |  | ACHE | CAV1 |
| PTGS2 | CDK2 |  | GSTP1 |  | NR3C2 | CYP1B1 |
| OPRM1 | RAF1 |  | CTSD |  | JUN | CDK2 |
| VEGFA | PRKCA |  | ADRA1B |  | GSTP1 | RAF1 |
| NFKBIA | PLAT |  | NPEPPS |  | ADRA1B | FASN |
| TNFAIP6 | PPARD |  | IL1A |  | DRD1 | PRKCA |
| MCL1 | CYP1A2 |  | SERPINE1 |  | LBP | PLAT |
| BIRC5 | ACP3 |  | SLPI |  |  | PPARD |
| CHRM3 | CHRM3 |  | GABRA1 |  |  | CYP1A2 |
| ADRB2 | BIRC5 |  | IKBKB |  |  | MAPK8 |
| SLC6A2 | GSTM1 |  | PCOLCE |  |  | ACP3 |
| APP | CLDN4 |  | MPO |  |  | BIRC5 |
| CD40LG | MYC |  | ADRA1A |  |  | CHRM3 |
| ESR1 | CXCL11 |  | CYP3A4 |  |  | GSTM1 |
| MAPK1 | ADH1C |  | MAPK14 |  |  | MTTP |
| ADH1C | SOD1 |  | LTA4H |  |  | CLDN4 |
| MMP8 | PPARG |  | ERBB3 |  |  | CXCL11 |
| RELA | THBD |  | CASP9 |  |  | MYC |
| PPARG | ALOX5 |  | CHRM1 |  |  | HMGCR |
| CHEK1 | GNRHR |  | IGF2 |  |  | SOD1 |
| PTGES | CHEK1 |  | PRKCB |  |  | PPARG |
| JUN | JUN |  | SCN5A |  |  | THBD |
| AKR1B1 | AKR1B1 |  | IL10RA |  |  | ALOX5 |
| GSK3B | PARP1 |  | MMP2 |  |  | CHEK1 |
| RB1 | ADRB1 |  | IL6R |  |  | JUN |
| IFNG | HSPB1 |  | AKT1 |  |  | AKR1B1 |
| ADRB1 | IGFBP3 |  | HMOX1 |  |  | GSK3B |
|  | CHUK |  | ERBB2 |  |  | SOAT1 |
|  | ABCG2 |  | SPP1 |  |  | PARP1 |
|  | MMP1 |  | CCNB1 |  |  | ADRB1 |
|  | STAT1 |  | POR |  |  | HSPB1 |
|  | PGR |  | INSRR |  |  | IGFBP3 |
|  | PSMD3 |  | CXCL10 |  |  | CHUK |
|  | RASSF1 |  | CAV1 |  |  | IL4 |
|  | KCNH2 |  | ADRA2A |  |  | ABCG2 |
|  | MMP3 |  | HAS2 |  |  | BAD |
|  | IL1B |  | CYP1B1 |  |  | MMP1 |
|  | F3 |  | CDK2 |  |  | STAT1 |
|  | CHEK2 |  | CCND1 |  |  | PGR |
|  | MAOB |  | RAF1 |  |  | PSMD3 |
|  | CXCL8 |  | DUOX2 |  |  | GOT1 |
|  | PRSS1 |  | NQO1 |  |  | RASSF1 |
|  | BCL2 |  | NCF1 |  |  | KCNH2 |
|  | TP53 |  | SLC6A3 |  |  | MMP3 |
|  | EGF |  | PTGS2 |  |  | IL1B |
|  | NR1I3 |  | VEGFA |  |  | F3 |
|  | MMP9 |  | PRKCA |  |  | CHEK2 |
|  | PPARA |  | NFKBIA |  |  | CXCL8 |
|  | GRM5 |  | PLAT |  |  | MAOB |
|  | ELK1 |  | CYP1A2 |  |  | PRSS1 |
|  | AR |  | PPARD |  |  | BCL2 |
|  | PTGS1 |  | CYP1A1 |  |  | ABCC1 |
|  | E2F2 |  | MAPK8 |  |  | TP53 |
|  | COL1A1 |  | TNFAIP6 |  |  | EGF |
|  | PLAU |  | VCAM1 |  |  | NR1I3 |
|  | RXRB |  | ACP3 |  |  | MMP9 |
|  | HTR3A |  | GSTM1 |  |  | PPARA |
|  | GJA1 |  | BIRC5 |  |  | OLR1 |
|  | GSTP1 |  | CASP8 |  |  | ELK1 |
|  | DRD1 |  | CHRM3 |  |  | AR |
|  | NPEPPS |  | CDK1 |  |  | PTGS1 |
|  | IL1A |  | ADRB2 |  |  | E2F2 |
|  | SLC6A4 |  | CCL2 |  |  | COL1A1 |
|  | PCOLCE |  | SLC6A2 |  |  | PLAU |
|  | MPO |  | NFE2L2 |  |  | RXRB |
|  | ERBB3 |  | CD40LG |  |  | HTR3A |
|  | CASP9 |  | CLDN4 |  |  | GJA1 |
|  | CHRM1 |  | MYC |  |  | GSTP1 |
|  | HMOX1 |  | CXCL11 |  |  | DRD1 |
|  | SPP1 |  | ESR1 |  |  | APOB |
|  | POR |  | BAX |  |  | NPEPPS |
|  | ERBB2 |  | PYGM |  |  | IL1A |
|  | CXCL10 |  | MAPK1 |  |  | IKBKB |
|  | ADRA2A |  | ADH1C |  |  | PCOLCE |
|  | HAS2 |  | HSPA5 |  |  | SLC6A4 |
|  | CCND1 |  | SOD1 |  |  | MPO |
|  | CACNA1S |  | RELA |  |  | ERBB3 |
|  | DUOX2 |  | PPARG |  |  | CASP9 |
|  | NQO1 |  | AHR |  |  | CHRM1 |
|  | NCF1 |  | THBD |  |  | HMOX1 |
|  | SLC6A3 |  | ALOX5 |  |  | SPP1 |
|  | PTGS2 |  | CHEK1 |  |  | POR |
|  | OPRM1 |  | JUN |  |  | ERBB2 |
|  | VEGFA |  | AKR1B1 |  |  | CXCL10 |
|  | NFKBIA |  | GSK3B |  |  | UGT1A1 |
|  | CYP1A1 |  | PARP1 |  |  | HAS2 |
|  | TNFAIP6 |  | RB1 |  |  | CCND1 |
|  | VCAM1 |  | ADRB1 |  |  | DUOX2 |
|  | GRIN2D |  | IFNG |  |  | NQO1 |
|  | CASP8 |  | COL3A1 |  |  | NCF1 |
|  | CDK1 |  |  |  |  | SLC6A3 |
|  | ADRB2 |  |  |  |  | PTGS2 |
|  | SLC6A2 |  |  |  |  | OPRM1 |
|  | CCL2 |  |  |  |  | VEGFA |
|  | NFE2L2 |  |  |  |  | NFKBIA |
|  | CD40LG |  |  |  |  | CYP1A1 |
|  | BAX |  |  |  |  | TNFAIP6 |
|  | ESR1 |  |  |  |  | VCAM1 |
|  | MAPK1 |  |  |  |  | CASP8 |
|  | DRD3 |  |  |  |  | DPEP1 |
|  | HSPA5 |  |  |  |  | ADRB2 |
|  | RELA |  |  |  |  | CDK1 |
|  | AHR |  |  |  |  | NFE2L2 |
|  | RB1 |  |  |  |  | SLC6A2 |
|  | IFNG |  |  |  |  | CCL2 |
|  | GABBR1 |  |  |  |  | CD40LG |
|  |  |  |  |  |  | ESR1 |
|  |  |  |  |  |  | BAX |
|  |  |  |  |  |  | PYGM |
|  |  |  |  |  |  | MAPK3 |
|  |  |  |  |  |  | MAPK1 |
|  |  |  |  |  |  | HSPA5 |
|  |  |  |  |  |  | RELA |
|  |  |  |  |  |  | FOSL2 |
|  |  |  |  |  |  | AHR |
|  |  |  |  |  |  | MAPK10 |
|  |  |  |  |  |  | RB1 |
|  |  |  |  |  |  | IFNG |
