# Supplementary figures and images for: Validation of the Anticolitis Efficacy of the Jian-Wei-Yu-Yang Formula
Source: Evid Based Complement Alternat Med. 2022 Aug 31;2022:9110704. doi: 10.1155/2022/9110704 (PMC9451982; doi:10.1155/2022/9110704)

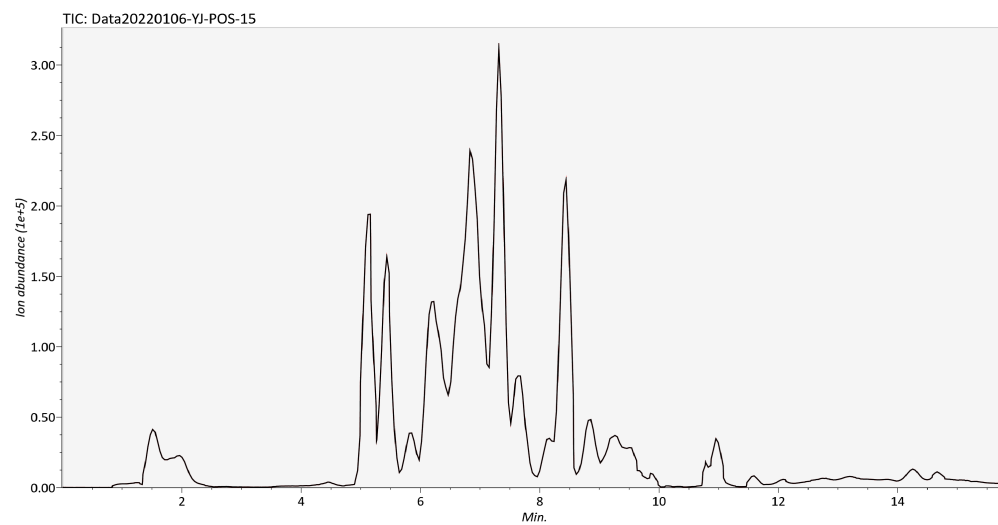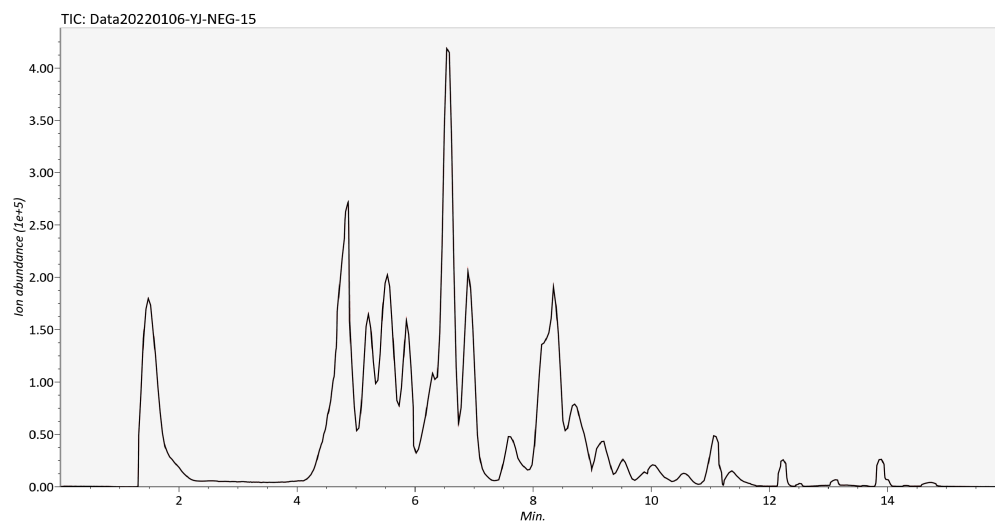

Supplement: Supplementary Materials — Supplementary Table 1: LC-MS/MS identified active components of JW. Supplementary Table 2: active components and targets of JW. Supplementary Table 3: differentially expressed genes between colorectal cancer patients and healthy cohorts. Supplementary Table 4: Lasso model of the JW target. Supplementary Table 5: alpha diversity indices of the gut microbiota after JW treatment. Supplementary Table 6: the altered metabolites in murine colitis models after JW treatment. Supplementary Figure 1: LC-MS/MS identified JW components. Supplementary Figure 2: the major active components of the JW formula. Supplementary Figure 3: microbial communities in colitis mice after JW treatment. Rank abundance curve (A), Shannon curves (B), rarefaction analysis (C), and the shared OTUs among three groups (D); BugBase predicts the phenotype of microbiota (E), and PICRUSt predicts the altered pathways after JW treatment (F). ∗∗∗p < 0.001 indicates a statistical difference from the DSS group. Supplementary Figure 4: MTT assay evaluating the toxicity of the JW serum. [file 9110704.f1.zip › sFig1.pdf]

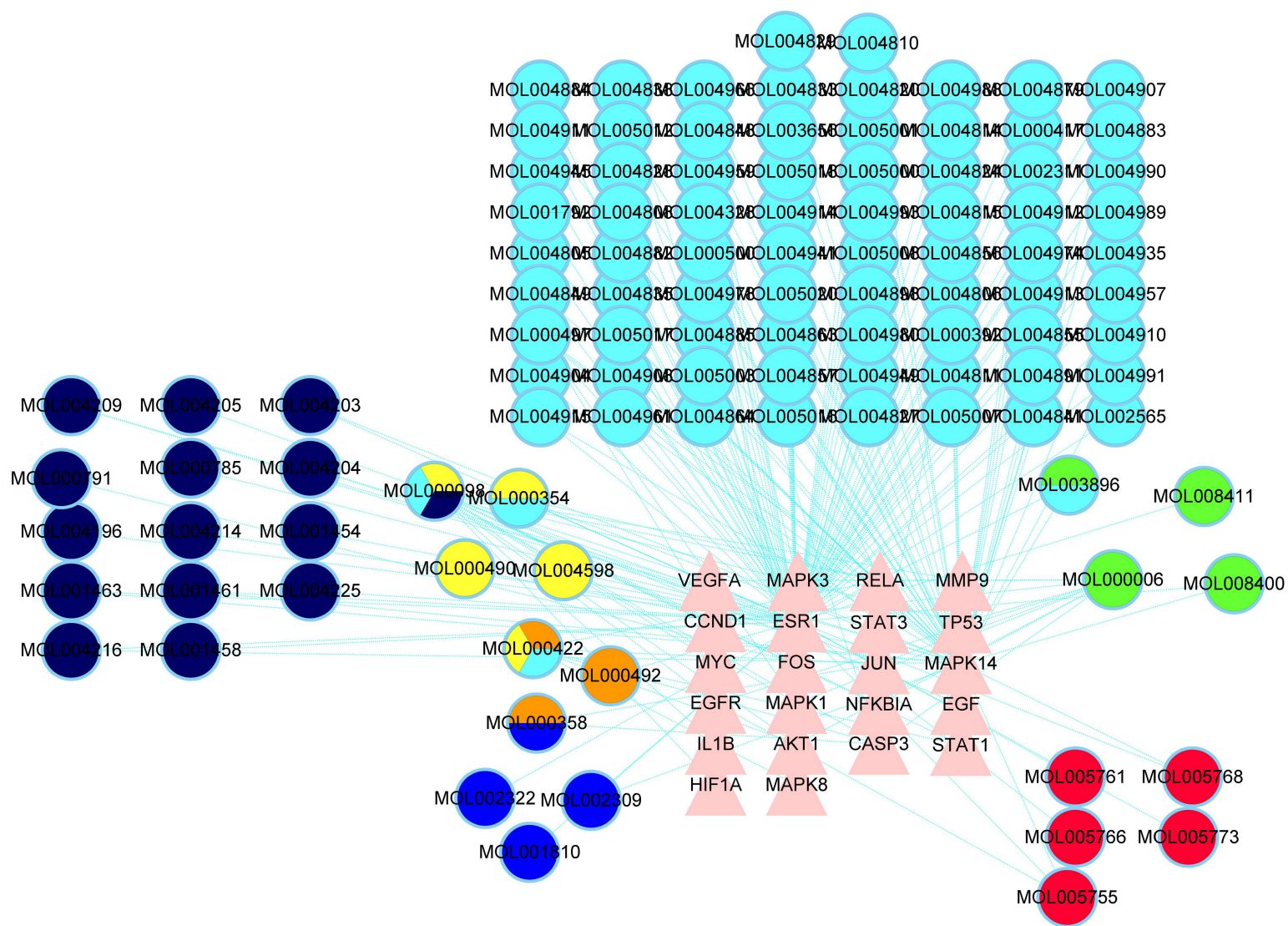

Supplement: Supplementary Materials — Supplementary Table 1: LC-MS/MS identified active components of JW. Supplementary Table 2: active components and targets of JW. Supplementary Table 3: differentially expressed genes between colorectal cancer patients and healthy cohorts. Supplementary Table 4: Lasso model of the JW target. Supplementary Table 5: alpha diversity indices of the gut microbiota after JW treatment. Supplementary Table 6: the altered metabolites in murine colitis models after JW treatment. Supplementary Figure 1: LC-MS/MS identified JW components. Supplementary Figure 2: the major active components of the JW formula. Supplementary Figure 3: microbial communities in colitis mice after JW treatment. Rank abundance curve (A), Shannon curves (B), rarefaction analysis (C), and the shared OTUs among three groups (D); BugBase predicts the phenotype of microbiota (E), and PICRUSt predicts the altered pathways after JW treatment (F). ∗∗∗p < 0.001 indicates a statistical difference from the DSS group. Supplementary Figure 4: MTT assay evaluating the toxicity of the JW serum. [file 9110704.f1.zip › sfig2.pdf]

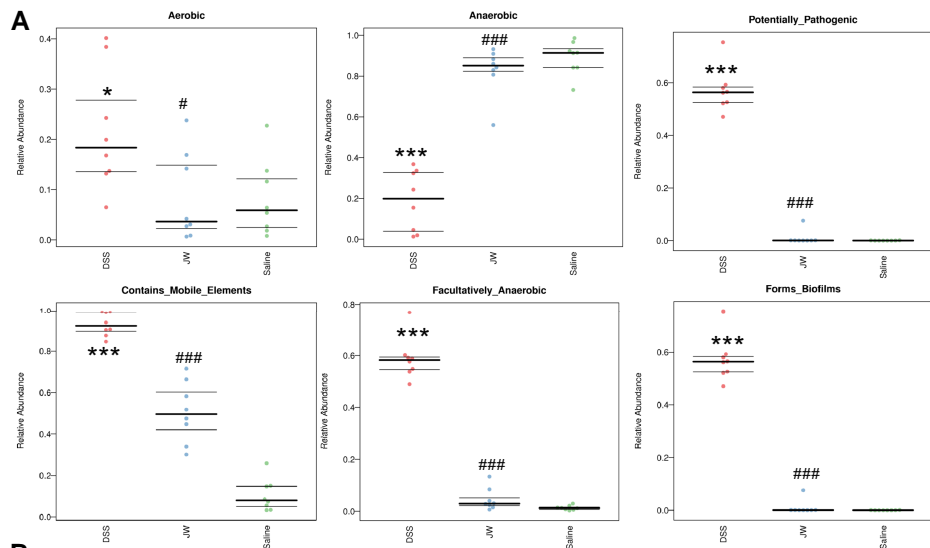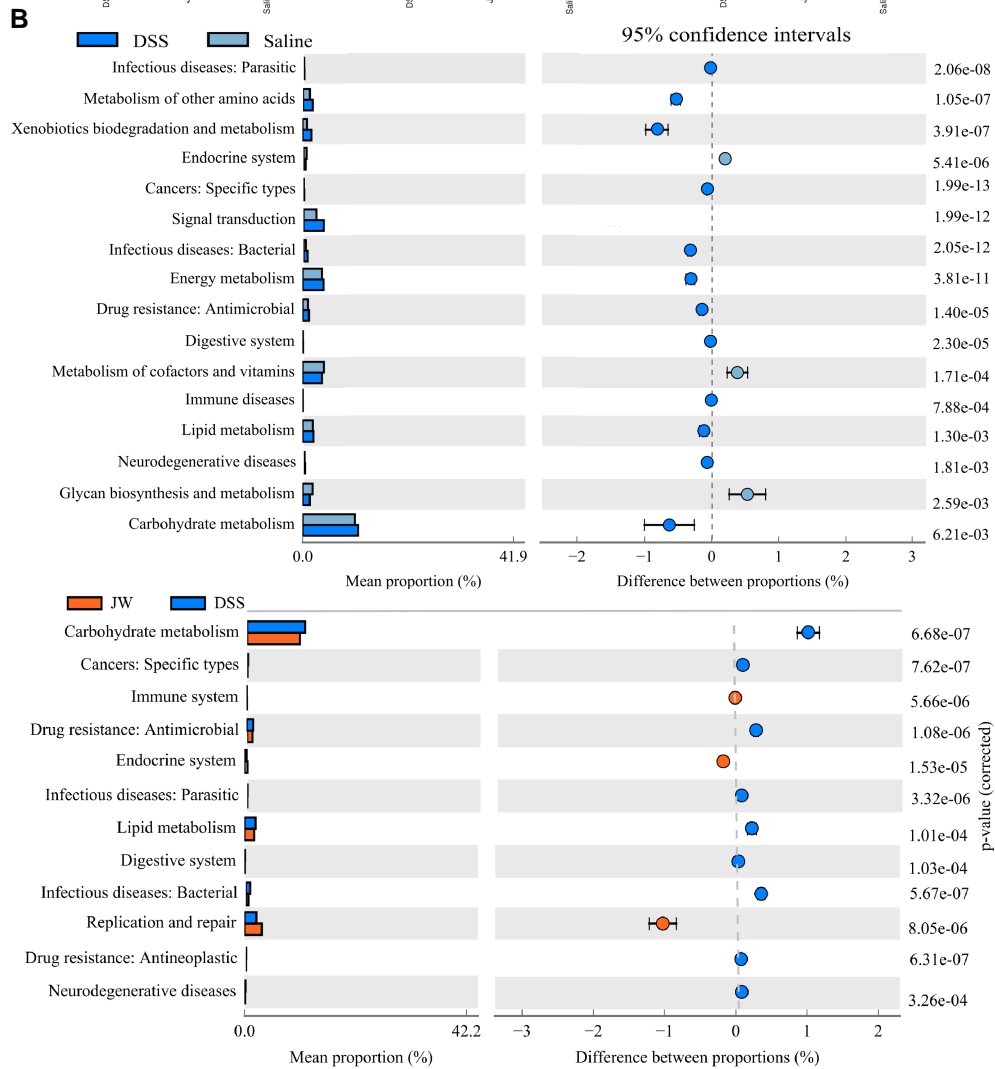

Supplement: Supplementary Materials — Supplementary Table 1: LC-MS/MS identified active components of JW. Supplementary Table 2: active components and targets of JW. Supplementary Table 3: differentially expressed genes between colorectal cancer patients and healthy cohorts. Supplementary Table 4: Lasso model of the JW target. Supplementary Table 5: alpha diversity indices of the gut microbiota after JW treatment. Supplementary Table 6: the altered metabolites in murine colitis models after JW treatment. Supplementary Figure 1: LC-MS/MS identified JW components. Supplementary Figure 2: the major active components of the JW formula. Supplementary Figure 3: microbial communities in colitis mice after JW treatment. Rank abundance curve (A), Shannon curves (B), rarefaction analysis (C), and the shared OTUs among three groups (D); BugBase predicts the phenotype of microbiota (E), and PICRUSt predicts the altered pathways after JW treatment (F). ∗∗∗p < 0.001 indicates a statistical difference from the DSS group. Supplementary Figure 4: MTT assay evaluating the toxicity of the JW serum. [file 9110704.f1.zip › sFig3.pdf]

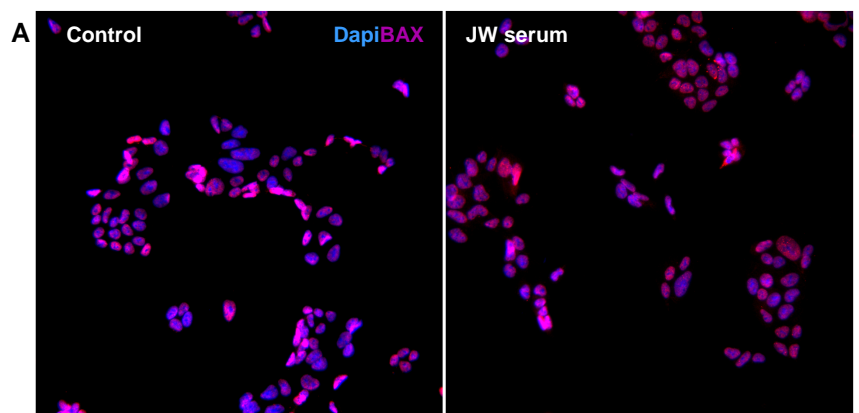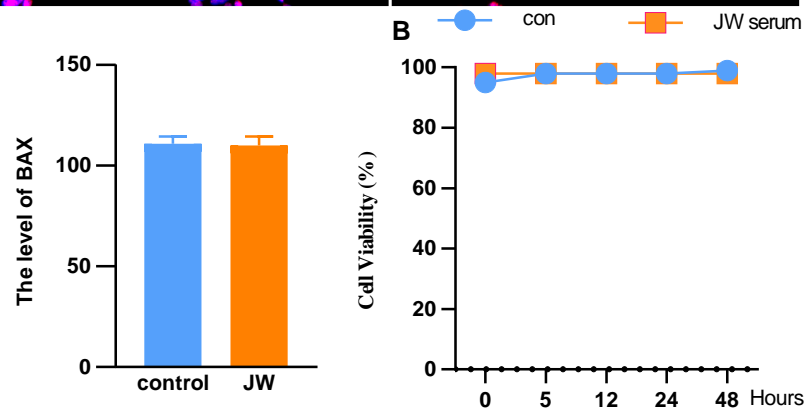

Supplement: Supplementary Materials — Supplementary Table 1: LC-MS/MS identified active components of JW. Supplementary Table 2: active components and targets of JW. Supplementary Table 3: differentially expressed genes between colorectal cancer patients and healthy cohorts. Supplementary Table 4: Lasso model of the JW target. Supplementary Table 5: alpha diversity indices of the gut microbiota after JW treatment. Supplementary Table 6: the altered metabolites in murine colitis models after JW treatment. Supplementary Figure 1: LC-MS/MS identified JW components. Supplementary Figure 2: the major active components of the JW formula. Supplementary Figure 3: microbial communities in colitis mice after JW treatment. Rank abundance curve (A), Shannon curves (B), rarefaction analysis (C), and the shared OTUs among three groups (D); BugBase predicts the phenotype of microbiota (E), and PICRUSt predicts the altered pathways after JW treatment (F). ∗∗∗p < 0.001 indicates a statistical difference from the DSS group. Supplementary Figure 4: MTT assay evaluating the toxicity of the JW serum. [file 9110704.f1.zip › sFig4.pdf]
